# Non-communicable disease (NCD) risk factors and diabetes among adults living in slum areas of Dhaka, Bangladesh

**DOI:** 10.1371/journal.pone.0184967

**Published:** 2017-10-03

**Authors:** Lal B. Rawal, Tuhin Biswas, Nusrat Nausheen Khandker, Shekhar Ranjan Saha, Mohammed Mahiul Bidat Chowdhury, Abdullah Nurus Salam Khan, Enamul Hasib Chowdhury, Andre Renzaho

**Affiliations:** 1 International Centre for Diarrhoeal Disease Research, Bangladesh (icddr,b), 68 Shaheed Tajuddin Ahmed Sharani, Mohakhali, Dhaka, Bangladesh; 2 James P Grant Schools of Public Health, BRAC University, Dhaka, Bangladesh; 3 The University of Queensland, Long Pocket Precinct, Indooroopilly Queensland, Australia; 4 Western Sydney University Locked Bag 1797, Penrith, NSW Australia; Swinburne University of Technology, AUSTRALIA

## Abstract

**Background:**

Despite one-third of the urban population in Bangladesh living in urban slums and at increased risk of non-communicable diseases (NCDs), little is known about the NCD risk profile of this at-risk population. The aim of the study was to identify the prevalence of the NCD risk factors and the association of NCD risk factors with socio-demographic factors among the adults of urban slums in Dhaka, Bangladesh.

**Method:**

A cross-sectional study was conducted among adult slum dwellers (aged 25 and above) residing in three purposively selected urban slums of Dhaka for at least six months preceding the survey. The risk factors assessed were- currently smoking, fruit and vegetable intake, physical activity, hypertension and body mass index (BMI). Information on self-reported diabetes was also taken. A total of 507 participants (252 females; 49.7%) were interviewed and their physical measures were taken using the WHO NCD STEPS instrument.

**Result:**

The overall prevalence of NCD risk factors was: 36.0% (95% CI: 31.82–40.41) for smoking; 95.60% (95% CI: 93.60–97.40) for insufficient fruit and vegetable intake; 15.30% (95% CI:12.12–18.71) for low physical activity;13.70% (95% CI: 10.71–16.92) for hypertension; 22.70% (95% CI: 19.31–26.02) for overweight or obesity; and 5.00% (95%: 3.20–7.00) for self-reported diabetes. In the logistic regression model, the clustering of three or more NCD risk factors was positively associated with younger age groups (p = 0.02), no formal education (p <0.001) and primary education level (p = 0.01), but did not differ by sex of the participants, monthly income and occupation.

**Conclusion:**

All NCD risk factors are markedly high among the urban slum adults. These findings are important to support the formulation and implementation of NCD-related polices and plan of actions that recognize urban slum populations in Bangladesh as a priority sub-population.

## Introduction

The global burden of non-communicable diseases (NCDs) is increasing rapidly as a result of a number of factors, such as economic development and related erosion of traditional food practices (increase in the intake of processed foods high in fat, salt and sugar) and change in cultural norms (increase in the use of tobacco and alcohol), decline in the physical activity, and increase in the sedentary lifestyles [1, 2]. These risk factors are often accelerated by rapid and unplanned urbanization [3, 4]. Globally, almost two-thirds of total deaths occur due to NCDs [1]. The 2014 NCD Global Status report showed that, of the 58 million deaths that occurred worldwide in 2012, 38 million were due to NCDs (almost two thirds), comprising mainly of cardiovascular diseases, cancers, diabetes and chronic lung diseases [1]. More than 40% of NCD deaths (16 million) were considered as premature deaths since they died before reaching average life span of 70 years. Almost three quarters of all NCD deaths (28 million) and the majority of premature deaths (82%), occurred in low and middle-income countries (LMICs).

NCD burden and associated inequalities will continue to increase in the next two decades. For example, while Asia and Africa still remain mostly rural (48% and 40% of their respective populations living in urban areas), these two regions are urbanizing faster than any other regions; and by 2050 it is projected that64% and 56% of their respective populations will be living in urban area [5]. Recent studies show that NCDs and their risk factors, especially body mass index and cholesterol levels rise rapidly in tandem with rapid urbanization [4, 6].

Like other LMICs, Bangladesh is also experiencing rapid urbanization. Urban population has increased exponentially, from21.3 million in 1990 to 53.1 million in 2014, and projected to reach 112.4 million by 2050 [5]. Almost one-third of the urban population lives in the slums, which are densely populated areas with poor housing and socio-economic conditions. This slum population most often lack the basic amenities required for urban life, putting them at risk of contracting and suffering from communicable as well as non-communicable diseases [7]. Rapid urbanization in Bangladesh brings with it the increased risks of NCDs. The estimated total number of deaths due to NCDs in Bangladesh in 2012 was over 886,000 which was almost two-third of total deaths[8].The majority of NCD deaths occurred in urban areas [9]. Although national surveillance and monitoring of NCDs in Bangladesh are yet to be established, a number of studies in the past have been conducted and they provide some level of evidence base in terms of the current situation of NCD burden in Bangladesh at national level and by urban and rural settings [10–12].Findings emerging from these studies suggest that all-cause mortality and NCDs and their risk factors such as hypertension and abdominal obesity are significantly higher in urban than rural areas [4, 13, 14].

However, there is still a lack of evidence base concerning the current situation of NCDs and their risk factors among the urban slum population in Bangladesh. Interestingly, awareness about NCD prevention and management remains poor in urban areas [3]. In this context, urban poor living in precarious slum conditions are confronted with an increased risk of both infectious diseases and the threat of NCDs, hence representing a double-whammy for both health professionals and policy makers. It is therefore important to better understand the true picture of NCD risk factors among the urban slum populations. This information is critical for the development of effective policies and program intervention aimed at improving healthy behavior and preventing the development of chronic diseases. Such interventions need to be based on formative research and tailored to the needs of the target population and the environments they live in. Therefore, the aim of the study was to establish the prevalence of selected NCD risk factors and identify association of NCD risk factors with known socio-demographic factors among the adults of urban slums in Dhaka, Bangladesh.

## Methods

### Study setting and population

A cross-sectional study was conducted in three purposively selected urban slums namely, Korail slum in Gulshan/Mahakali, Bhashantek slum in Mirpur and Rayar Bazar slum in Dhanmondi of Dhaka. Adult slum dwellers (aged 25 to 64 years) residing in those areas for at least six months preceding the survey were included in the study. WHO recommends the use of this age group for the study of NCD risk factors [15]. Critically ill or bed-ridden adults, pregnant women and people with physical or mental disability were excluded from the survey.

### Sampling strategy and study samples

A household (e.g. a unit consisting of one or more people who live in the same dwelling and share meal) was the sampling unit. A household was identified using a multi-stage cluster sampling technique. In the first stage, maps of the three purposively selected urban slums (Korail, Bhashantek and Rayar Bazar slum areas) were established and geographical clusters in each slum were identified based on the road networks. In the second stage, the clusters were divided into equal portions—six from one slum and four each from two slums, with total clusters of 14 were generated. Real time images of each cluster were obtained by using Google Earth and used as guides for sampling. In the final stage, two clusters in each slum were selected by using simple random sampling and Global Positioning Systems (GPS) co-ordinates of each clusters were noted. The households within each cluster were selected using a systematic random sampling. The south-east co-ordinate point was used as the starting point and sampling commenced following right-hand movement. Every second households were approached and male and female respondents were also chosen from these households in an alternate manner. If there were more than one eligible member within a household, one was selected randomly using lottery. Finally,519 eligible participants provided information along with measurements of necessary physical measures. Twelve samples which had incomplete information were then excluded from the analyses following the WHO STEPS guidelines. Further, four samples which did not present any NCD risk factors, which was the main outcome measure of this study, were then excluded from the final analyses.

### Data collection process

The study data was collected during the month of November 2015, through face-to face interviews, using an interviewer assisted structured questionnaire. The questionnaire was adopted from the Step I [16] and Step II [17] of the WHO STEPS Questionnaire. Socio-demographic information such as age and gender, education, marital and work status was collected. The data on NCD risk factors including tobacco use, fruit and vegetable consumption, physical activity, and history of chronic conditions were collected by trained interviewers. The data collection team included five research team members (including two physicians) and eight trained field research assistants with a health science background. Data collectors were exclusively trained before going to the field, by verbal instructions, demonstrations, mock interviews and field pilot testing. Among the data collectors, there were persons who were skilled in taking anthropometric measurements and measuring blood pressure. The research team additionally trained them so that the measurements were in accordance with the WHO guidelines. At first, the data collectors conducted interviews under the supervision of research team members and later, on their own with occasional scrutiny from the researchers. At the end of each day, random checks of the interviews were performed to identify any non-response pattern or errors in data collection. These findings were conveyed to the data collectors through group meeting and individual feedbacks were given the next day.

At each location, once the designated researcher initiated the sampling frame by choosing a household and whether to sample a male or a female from that household, two data collectors commenced the interview—one conducted the interview while the other took anthropometric measurements and recording of blood pressure. If an eligible member of the required gender could not be located at the household or if met with refusal to participate, the data collectors report this to the researcher, who then moved along to the next household on the sampling frame. The Bangla version of questionnaire which was already been used in the previous NCD risk factor study in Bangladesh was used with minor changes in wordings and language that suit in the slum area context. The revised questionnaire was then reviewed by the research team members and experts before the pilot testing at different slum than the selected study areas. The comments and feedback received from the expert review and field pilot testing was incorporated and the final pilot tested Bangla version questionnaire was used for data collection.

### Outcome variables and their measurement

In total, six NCD risk factors namely current smoker, insufficient intake of fruits and vegetable, insufficient physical activity, currently hypertensive, body mass index(BMI) status, and currently diabetic were assessed as the outcome variables for this study.

#### Current smoker

Questions were asked to identify current smokers (including those who had smoked in the past 30 days). Pictorials showing the various tobacco products were used as cue cards as per WHO recommendations [18].

#### Intake of fruit and vegetable

Information was recorded on the number of days that respondents consumed fruits and vegetables in a typical week, and the number of servings of fruits and vegetables consumed on average per day. The WHO food frequency questionnaire was used. As recommended by WHO [18], the consumption of less than five servings of fruits and vegetables per day was classified as insufficient fruit and vegetable intake.

#### Physical activity

Physical activity was assessed using the Global Physical Activity Questionnaire (GPAQ) [19, 20]. The GPAQ asks respondents about activities for transport purposes, vigorous and moderate activity at work, and vigorous and moderate activity in leisure time, and time spent sitting. Show-cards with culturally relevant examples were used to aid respondents in classifying activities. Analysis and categorization followed existing guidelines [21]. Those who did not meet the criteria for vigorous and moderate intensity activities (less than 75 minutes of vigorous-intensity physical activity or less than 150 minutes of moderate intensity physical activity per week or equivalent of combinations of these (from work, walking/cycling and leisure) were categorized as having insufficient physical activity.

#### Measurement of blood pressure

Blood pressure was measured using a digital, automated blood pressure monitor (OMRON digital device, OMRON, Netherlands) with an appropriate sized cuff with the participant in sitting position and while taking rest at their house. Trained research assistants measured blood pressure twice at two-minute interval and the average of the two measurements was used in the analysis. Using the criteria recommended by WHO [17, 22], hypertension was defined as having systolic blood pressure of 140 mm Hg and/or diastolic blood pressure of 90 mm Hg or high during the study, or being previously diagnosed hypertensive. Previously diagnosed hypertension was determined documenting a treatment record book, or participant’s history of medication use for hypertension.

#### Measurement of BMI

Using the STEPS protocol and recommended instruments ensured accuracy of height, weight and blood pressure measurements [17, 18]. Height was recorded in centimeters using a portable standard stature measuring board. Weight was recorded in kilograms using a portable digital weighing scale (Seca, Germany). Digital weighing scales was placed on a flat surface at first and weight was measured twice with light clothing on and without shoes with precision of 0.1 kg. The average of the two measurements was used in the analysis. Both weight and height were measured at the participant’s home. Next, body mass index (BMI) was calculated using this formula: BMI = weight in kg/height in sq. meter. Using WHO classification for obese and overweight, a BMI of 30 and above was considered as obese and the BMI between 25.0 and 29.9 was considered as overweight.

#### Assessment of diabetic status

Self-reported information was collected from the participants to identify whether they are diabetic or not. At first the data collectors asked the participants if they were ‘diabetic’, if the respondent said ‘yes’ then they were asked to show the prescription for validating if they were taking any medication or asked to show regular medicines that they take. The physicians in the research team cross-checked the prescription or drugs for presence of any glucose lowering medication. In other case, where the respondent failed to report of their diabetic status or could not show any relevant evidence, he or she was not considered as being diabetic.

### Data analysis

The data analysis is performed among the total samples of 507 participants. Of 519 survey questionnaires, 12 were excluded which had incomplete information. The prevalence of selected NCD risk factors was estimated as percentage and 95% CIs were calculated. Chi-square statistics were used to test associations between covariates and risk factors. Logistic regression model was performed to assess the determinants of NCD risk factors. In adjusted logistic regression model, age, sex, occupation, education, family size and monthly income were adjusted. Logistic regression model was used to assess the effect of exposure variables on the outcome measures, adjusted for age, sex, occupation, highest level of education, and monthly income. Data was analyzed using the statistical software, IBM SPSS Statistics (version 20). P-valueless than 0.05 was considered as statistically significant for any of the statistical tests performed.

### Ethics approval

Ethics approval was obtained from the Ethical Review Committee of James P Grant School of Public Health, BRAC University, Bangladesh. Participants were recruited using a plain language statement in Bangla and verbal consent was obtained prior to the interview and data collection commencing. Due to the contextual facts that most participants were illiterate as well as the sensitive nature of requesting thumbprints or signatures, verbal informed consent was taken from the participants prior to the interview and data collection. The consent script was read out clearly by the interviewer and participants’ consent to participate or not to participate in the study was recorded.

## Results

### Participants’ characteristics

We present the socio-demographic characteristics of 507 participants in **Table 1** according to the number of listed risk factors they had. The mean age and ±SD of the participants was 37.8 (±11.1) years, with male and female ratio almost equal. The majority of the participants were non-manual worker (69.8%) and over half (51.5%) had no history of schooling. Almost a-third (30.4%) had monthly family income of less than or equal to 7,500 BDT (equal to 95 USD in year 2015). Overall prevalence of participants having at least one NCD risk factor was 19.5%, two NCD risk factors 49.3%, three NCD risk factors 25.3% and four or more 5.1%. Four participants (all males and below the age 44 years old) had no NCD risk factor (0.8%).

**Table 1:** Socio-demographic characteristics of the study participants according to number of listed risk factors for NCD.

General characteristics	No risk factor (n = 4)	1 risk factor (n = 99)	2 risk factors (n = 250)	3 risk factors (n = 128)	4 or more risk factors (n = 26)	Total (n = 507)	p-value
**Age group**							
25–34	4 (100)	57 (57.6)	114 (45.6)	45 (35.2)	6 (23.1)	226 (44.6)	<0.10
35–44	0 (0)	22 (22.2)	72 (28.8)	35 (27.3)	9 (34.6)	138 (27.2)	
45–54	0 (0)	12 (12.1)	35 (14.0)	27 (21.1)	4 (15.4)	78 (15.4)	
55–64	0 (0)	8 (8.1)	29 (11.6)	21 (16.4)	7 (26.9)	65 (12.8)	
**Sex**							
Male	2 (50)	40 (40.4)	137 (54.8)	65 (50.8)	11 (42.3)	255 (50.3)	0.87
Female	2 (50)	59 (59.6)	113 (45.2)	63 (49.2)	15 (57.7)	252 (49.7)	
**Marital status**							
Married	4 (100)	89 (89.8)	236 (94.4)	116 (90.6)	20 (76.9)	465 (91.7)	<0.001
Others	0 (0)	10 (10.2)	14 (5.6)	12 (9.4)	6 (23.1)	42 (8.3)	
**Occupation**							
Non-manual ^a^	2 (50)	81 (81.8)	165 (66.0)	83 (64.8)	23 (88.5)	354 (69.8)	<0.001
Manual	2 (50)	18 (18.2)	85 (34.0)	45 (35.2)	3 (11.5)	153 (30.2)	
**Highest level of education**							
No schooling	2 (50)	39 (39.4)	127 (50.8)	77 (60.2)	16 (61.5)	261 (51.5)	<0.001
less than primary school	0 (0)	15 (15.2)	44 (17.6)	25 (19.5)	2 (7.7)	86 (17.0)	
Primary school completed	0 (0)	14 (14.1)	29 (11.6)	14 (10.9)	5 (19.2)	62 (12.2)	
Secondary school and higher	2 (50)	31 (31.3)	50 (20.0)	12 (9.4)	3 (11.5)	98 (19.3)	
**Total family member**							
≤ 4 family members	1 (25)	56 (56.6)	107 (43.3)	56 (44.1)	13 (50.0)	233 (46.3)	0.11
≥ 5 family members	3 (75)	43 (43.4)	140 (56.7)	71 (55.9)	13 (50.0)	270 (53.7)	
**Household income (in BDT** ^b^)							
≤ 7500 (≤ 95 USD)	2 (50)	23 (23.7)	81 (32.5)	35 (27.6)	12 (46.2)	153 (30.4)	0.33
7501 to 10000 (95 to 127 USD)	0 (0)	20 (20.6)	60 (24.1)	32 (25.2)	5 (19.2)	117 (23.3)	
10001 to 15000 (127 to 191 USD)	1 (25)	30 (30.9)	51 (20.5)	26 (20.5)	5 (19.2)	113 (22.5)	
Above 15000 (above 191 USD)	1 (25)	24 (24.7)	57 (22.9)	34 (26.8)	4 (15.4)	120 (23.9)	
**Overall prevalence**	**4 (0.8)**	**99 (19.5)**	**250 (49.3)**	**128 (25.3)**	**26 (5.1)**	**507 (100)**	

Note: Chi-square tests were performed without selecting the first column (no risk factor n = 4)

^a^ Non-manual category includes sedentary workers, professionals, housewives, currently retired or unemployed.

^b^ BDT- Bangladeshi Taka

### Prevalence of NCD risk factors

**Table 2** presents findings on prevalence of NCD risk factors according to the selected socio-demographic characteristics of the participants as below:

#### Currently smoking

Over a third (35.7%, 95% CI: 31.82–40.41) adults in slum areas said that they smoked within the past 30 days preceding the study, with the highest proportion among those between 55 and 64 years old (49.2%). Almost three quarters men (71.1%) smoked, which rate was almost none for women (0.4%). Smoking rate was three times higher among manual workers (67.5%) compared to those of non-manual workers (22.4%), and higher (50.0%) among those who had below primary education level (50.0%).

#### Intake of fruits and vegetable

Almost all adults (95.6%, 95% CI: 93.60–97.40) responded that they had insufficient fruit and vegetable intake (using WHO recommendations) and by sex the proportion did not differ (males 93.7% and females 96.0%).

#### Physical activity

Using WHO recommendations for required duration of physical activity, those who did not meet the WHO recommendations was 15.3% (95% CI: 12.12–18.71) with higher proportion among adults of 55–64 years old (21.5%). Compared to males, females proportion for low physically activity was slightly higher (13.0% vs. 17.6%). This proportion was higher among the non-manual workers (18.2%), compared to the manual workers (8.6%).

#### Blood pressure

Overall, 13.7% (95% CI: 10.71–16.92) adults had high blood pressure (BP ≥ 140/90 mmHg), with a high proportion among adults of 45–64 years’ age group (26.9%). Those households having income of more than BDT 15,000 per month was 18.5% and this proportion was slightly higher among the females (14.8%) compared to the males (12.6%).

#### Body mass index

Body mass index ≥25 kg/m^2^ was considered as being overweight and obesity. Overall, it accounted 22.7% (95% CI: 19.30–26.40) and almost a quarter adult (23.1%) aged 45–54 years old were overweight or obese. This proportion was higher among females (30.4%) and non-manual workers (23.9%). Those who were underweight was 19.2% (BMI<18.50 as recommended by WHO).

#### Diabetes

Using the self-reported information, the overall prevalence of diabetes was 5.0% (95% CI: 3.20–7.00). The proportion was higher among those of 55–64 years’ age groups (7.7%), females (6.0%) and those having income more than BDT 15,000 per month.

**Table 2:** NCD risk factors by selected socio-demographic characteristics (n = 507).

Variables	Currently smoking	Fruits & Vegetables intake	Insufficient PA ^a^ (n = 77)	Hypertension (BP ≥ 140/90 mmHg)	Overweight or obesity	Diabetic
	n (%)	n (%)	n (%)	n (%)	n (%)	n (%)
**Overall prevalence**	**181 (35.7)**	**481 (95.6)**	**77 (15.3)**	**69 (13.7)**	**114 (22.7)**	**25 (5.0)**
**Age group**	** **	** **	** **			
25–34	78 (35.1)	211 (95.0)	32 (14.4)	16 (7.2)	56 (25.2)	9 (4.1)
35–44	47 (34.1)	136 (98.6)	20 (14.5)	17 (12.3)	28 (20.3)	7 (5.1)
45–54	24 (30.8)	72 (92.3)	11 (14.1)	21 (26.9)	18 (23.1)	4 (5.1)
55–64	32 (49.2)	62 (95.4)	14 (21.5)	15 (23.1)	12 (18.5)	5 (7.7)
**Sex **	** **	** **	** **	** **	** **	** **
Male	180 (71.1)	239 (94.5)	33 (13)	32 (12.6)	38 (15)	10 (4)
Female	1 (0.4)	242 (96.8)	44 (17.6)	37 (14.8)	76 (30.4)	15 (6)
**Occupation**	** **	** **	** **			
Non-manual	79 (22.4)	338 (96.0)	64 (18.2)	54 (15.3)	84 (23.9)	17 (4.8)
Manual	102 (67.5)	143 (94.7)	13 (8.6)	15 (9.9)	30 (19.9)	8 (5.3)
**Education level**	** **	** **	** **			
No schooling	80 (30.9)	252 (97.3)	44 (17)	38 (14.7)	64 (24.7)	12 (4.6)
Below primary school level	43 (50.0)	80 (93.0)	11 (12.8)	11 (12.8)	15 (17.4)	5 (5.8)
Primary school completed	22 (35.5)	60 (96.8)	12 (19.4)	7 (11.3)	19 (30.6)	12 (4.6)
Secondary school and higher	36 (37.5)	89 (92.7)	10 (10.4)	13 (13.5)	16 (16.7)	12 (4.6)
**Number of family member**	** **	** **	** **			
≤ 4 family members	83 (35.8)	219 (94.4)	35 (15.1)	30 (12.9)	51 (22)	15 (6.5)
≥ 5 family members	98 (36.2)	262 (96.7)	42 (15.5)	39 (14.4)	63 (23.2)	10 (3.7)
**Monthly income (in BDT**^**b**^**)**	** **	** **	** **			
≤ 7500 (≤ 95 USD)	42 (27.8)	148 (98.0)	27 (17.9)	16 (10.6)	42 (27.8)	8 (5.3)
7501 to 10000 (95 to 127 USD)	52 (44.4)	115 (98.3)	13 (11.1)	15 (12.8)	25 (21.4)	4 (3.4)
10001 to 15000 (127 to 191 USD)	43 (38.4)	98 (87.5)	20 (17.9)	16 (14.3)	23 (20.5)	4 (3.6)
Above 15000 (above 191 USD)	44 (37.0)	116 (97.5)	16 (13.4)	22 (18.5)	24 (20.2)	9 (7.6)

^**a**^ Insufficient PA -Insufficient physical activity

^**b**^ BDT- Bangladeshi Taka

### Factors associated with NCD risk

The unadjusted and adjusted odds ratio (OR) for the determinants of NCD risk factors is presented in **Table 3 and Table 4**. The older age slum dwellers (55–64 years) had higher odds (UOR: 1.79, 95% CI: 1.02–3.13) of smoking compared to the younger age group (25–34 years) and was statistically significant (p = 0.04). After adjusting for potential confounders, this was not statistically significant. There was a positive association between the age and the likelihood of having high BP (p<0.001) in both unadjusted and adjusted for covariates. The odds of females being overweight or obese was over three times higher (adjusted OR: 3.37, 95% CI: 1.84–6.15) and was statistically significant (p<0.001).

**Table 3:** Association between socio-demographic characteristics and selected NCD risk factors (currently smoking, fruits and vegetables consumption, and physical activity) (Unadjusted odds ratios (UOR) and adjusted odds ratio (AOR) are estimated using regression model) (n = 507).

Variables	Currently smoking		Fruits &Vegetables <5 servings		Insufficient PA ^c^	
	UOR ^a^ (95% CI)	AOR ^b^ (95% CI)	UOR (95%CI)	AOR (95% CI)	UOR (95% CI)	AOR (95% CI)
**Age group**						
25–34	**Ref **	** Ref**	**Ref**	**Ref**	**Ref**	**Ref**
35–44	0.95 (0.61–1.49)	0.77 (0.36–1.65)	3.55 (0.77–16.24)	2.88 (0.59–14.08)	1.01 (0.55–1.84)	1.11 (0.58–2.14)
45–54	0.82 (0.47–1.43)	0.49 (0.22–1.11)	0.63 (0.22–1.75)	0.43 (0.13–1.36)	0.97 (0.47–2.04)	1.04 (0.48–2.28)
55–64	1.79 (1.02–3.13)*	0.97 (0.40–2.35)	1.08 (0.29–3.98)	0.64 (0.14–2.82)	1.63 (0.81–3.28)	1.71 (0.78–3.71)
**Sex**						
Male	**Ref **	** Ref**	**Ref**	**Ref**	**Ref**	**Ref**
Female			1.77 (0.73–4.30)	1.42 (0.44–4.46)	1.42 (0.87–2.32)	0.91 (0.49–1.69)
**Occupation**						
Non-manual	**Ref **	** Ref**	**Ref**	**Ref**	**Ref**	**Ref**
Manual	7.19 (4.71–10.98)	1.41 (0.75–2.61)	0.74 (0.30–1.82)	0.92 (0.31–2.84)	0.75 (0.27–2.04)	2.69 (1.28–5.65)
**Highest level of education**						
No formal education	0.74 (0.46–1.22)	1.81 (0.79–4.09)	2.83 (0.97–8.32)	1.96 (0.56–6.82)	1.76 (0.85–3.65)	1.95 (0.86–4.42)
Below primary level	1.67 (0.92–3.01)	1.53 (0.64–3.64)	1.05 (0.34–3.25)	1.07 (0.30–3.74)	1.26 (0.51–3.14)	1.61 (0.62–4.13)
Primary Education	0.92 (0.47–1.78)	1.41 (0.51–3.93)	2.36 (0.47–11.75)	1.11 (0.19–6.32)	2.06 (0.83–5.12)	2.34 (0.90–6.04)
Secondary Education and above	**Ref **	** Ref**	**Ref**	**Ref**	**Ref**	**Ref**
**Total family member**						
≤ 4 family members	**Ref **	** Ref**	**Ref**	**Ref**	**Ref**	**Ref**
≥ 5 family members	1.02 (0.71–1.47)	1.04 (0.57–1.88)	1.73 (0.72–4.12)	2.09 (0.78–5.59)	1.03 (0.63–1.68)	0.84 (0.5–1.42)
**Monthly income (in BDT** ^d^**)**	** **	** **	** **	** **		
≤ 7500 (≤ 95 USD)	0.66 (0.39–1.1)	0.72 (0.32–1.62)	1.28 (0.25–6.44)	0.99 (0.18–5.38)	1.41 (0.72–2.74)	1.37 (0.67–2.82)
7501 to 10000 (95 to 127 USD)	1.36 (0.81–2.30)	1.12 (0.49–2.44)	1.49 (0.24–9.07)	1.36 (0.22–8.65)	0.80 (0.37–1.76)	0.94 (0.42–2.11)
10001 to 15000 (127 to 191 USD)	1.06 (0.62–1.81)	1.19 (0.51–2.76)	0.18 (0.05–0.65)*	0.15 (0.04–0.57)	1.4 (0.68–2.86)	1.68 (0.80–3.54)
Above 15000 (above 191 USD)	**Ref **	** Ref**	**Ref**	**Ref**	**Ref**	**Ref**

^a^ UOR: Unadjusted odds ratio

^b^ AOR: Adjusted odds ratio; adjusted for age, sex, occupation, education, family size and monthly income

^**c**^ Insufficient PA -Insufficient physical activity

^d^ BDT- Bangladeshi Taka

* p<0.05

**Table 4:** Association between socio-demographic characteristics and selected NCD risk factors (hypertension, overweight/obesity and diabetes) (Unadjusted odds ratios (UOR) and adjusted odds ratio (AOR) are estimated using regression model (n = 507).

Variables	Hypertension (BP ≥ 140/90 mmHg)		Overweight or obesity		Diabetes	
	UOR ^a^ (95% CI)	AOR ^b^ (95% CI)	UOR (95% CI)	AOR (95% CI)	UOR	AOR (95% CI)
**Age group**						
25–34	**Ref **	** Ref**	**Ref**	**Ref**	**Ref**	**Ref**
35–44	1.81 (0.88–3.71)	2.08 (0.97–4.46)	0.75 (0.45–1.26)	0.74 (0.40–1.31)	1.26 (0.46–3.48)	1.35 (0.46–3.94)
45–54	4.74 (2.32–9.68)**	5.58 (2.60–11.91)**	0.89 (0.48–1.63)	0.97 (0.50–1.87)	1.28 (0.38–4.28)	1.57 (0.45–5.53)
55–64	3.86 (1.79–8.34)**	4.94 (2.11–11.54)**	0.67 (0.33–1.35)	0.82 (0.38–1.74)	1.97 (0.64–6.11)	2.76 (0.81–9.45)
**Sex **						
Male	**Ref **	** Ref**	**Ref**	**Ref**	**Ref**	**Ref**
Female	1.2 (0.72–2.11)	1.3 (0.67–2.53)	2.47 (1.60–3.83)**	3.37 (1.84–6.15)**	1.55 (0.68–3.52)	2.67 (0.88–8.13)
**Occupation**						
Non-manual	**Ref **	** Ref**	**Ref**	**Ref**	**Ref**	**Ref**
Manual	0.61 (0.33–1.12)	0.65 (0.31–1.37)	0.79 (0.49–1.26)	1.65 (0.87–3.13)	1.1 (0.47–2.61)	2.15 (0.68–6.81)
**Highest level of education**						
No formal education	1.1 (0.56–2.16)	1.06 (0.48–2.34)	1.64 (0.89–3.01)	1.42 (0.71–2.77)	1.12 (0.35–3.55)	1.07 (0.29–3.92)
Below primary level	0.94 (0.4–2.22)	1.1 (0.44–2.76)	1.06 (0.49–2.29)	1.10 (0.49–2.51)	1.42 (0.37–5.47)	1.7 (0.41–7.06)
Primary Education	0.81 (0.3–2.17)	0.93 (0.32–2.66)	2.21 (1.03–4.73)*	1.95 (0.86–4.39)	1.59 (0.38–6.59)	1.91 (0.43–8.55)
Secondary Education and above	**Ref **	** Ref**	**Ref**	**Ref**	**Ref**	**Ref**
**Total family member**						
≤ 4 family members	**Ref **	** Ref**	**Ref**	**Ref**	**Ref**	**Ref**
≥ 5 family members	1.13 (0.68–1.89)	0.81 (0.46–1.42)	1.07 (0.71–1.64)	1.04 (0.66–1.64)	0.554 (0.24–1.25)	0.46 (0.2–1.10)
**Monthly income (in BDT** ^**c**^**)**						
≤ 7500 (≤ 95 USD)	0.52 (0.26–1.05)	0.49 (0.23–1.05)	1.53 (0.86–2.70)	1.15 (0.62–2.14)	0.68 (0.26–1.83)	0.52 (0.18–1.51)
7501 to 10000 (95 to 127 USD)	0.65 (0.32–1.32)	0.79 (0.37–1.67)	1.08 (0.57–2.02)	1.04 (0.54–2.02)	0.43 (0.13–1.45)	0.37 (0.11–1.33)
10001 to 15000 (127 to 191 USD)	0.73 (0.36–1.48)	0.79 (0.38–1.66)	1.02 (0.54–1.94)	0.94 (0.48–1.84)	0.45 (0.14–1.51)	0.41 (0.12–1.43)
Above 15000 (above 191 USD)	**Ref **	** Ref**	**Ref**	**Ref**	**Ref**	**Ref**

^a^ UOR: Unadjusted odds ratio

^b^ AOR: Adjusted odds ratio; adjusted for age, sex, occupation, education, family size and monthly income

^c^ BDT- Bangladeshi Taka

* p<0.05

** p<0.001

### Clustering the NCD risk factors

**Table 5** presents findings by clustering the NCD risk factors one vs. two risk factors and one vs. three or more risk factors and identifies the association with socio-demographic characteristics. The unadjusted and adjusted odds ratios are presented using logistic regression model. After adjusting for potential confounding factors, three or more NCD risk factors were positively associated with the age and education (See Table 5)

**Table 5:** Association between socio-demographic characteristics and clustering of NCD risk factors using logistic regression model (n = 503).

Variables	1 risk factor vs. ≥ 2 risk factors		1 risk factor vs. ≥ 3 risk factors	
	UOR ^a^ (95% CI)	AOR ^b^ (95% CI)	UOR (95% CI)	AOR (95% CI)
**Age group**		** **	** **	** **
25–34	**Ref**	**Ref**	**Ref**	**Ref**
35–44	1.64 (0.92–2.91)	1.39 (0.57–3.36)	2.24 (1.18–4.22)*	2.87 (1.15–7.18)*
45–54	1.46 (0.7–3.02)	1.44 (0.66–3.13)	2.89 (1.34–6.21)*	2.75 (1.21–6.24)*
55–64	1.81 (0.78–4.22)	1.42 (0.77–2.64)	3.91 (1.64–9.35)**	1.78 (0.9–3.52)
**Sex**		** **	** **	** **
Female	**Ref**	**Ref**	**Ref**	**Ref**
Male	1.79 (1.11–2.87)*	1.6 (0.89–2.88)	1.44 (0.86–2.41)	1.29 (0.67–2.49)
**Occupation**		** **	** **	** **
Non-manual	**Ref**	**Ref**	**Ref**	**Ref**
Manual	2.32 (1.31–4.12)**	1.7 (0.83–3.46)	2.04 (1.12–3.77)*	1.55 (0.72–3.36)
**Highest level of education**		** **	** **	** **
No formal education	2.02 (1.14–3.58)*	1.78 (0.93–3.43)	4.93 (2.41–10.13)**	4.07 (1.83–9.05)**
Below primary level	1.82 (0.87–3.81)	1.48 (0.68–3.23)	3.72 (1.54–8.99)**	3.22 (1.28–8.14)*
Primary education completed	1.28 (0.59–2.80)	1.11 (0.49–2.52)	2.8 (1.11–7.08)*	2.61 (0.99–6.85)
Secondary education and above	**Ref**	**Ref**	**Ref**	**Ref**
**Monthly income (in BDT** ^**c**^**)**		** **	** **	** **
≤ 7,500 (≤ 95 USD)	**Ref**	**Ref**	**Ref**	**Ref**
7,501–10,000 (95 to 127 USD)	0.85 (0.43–1.69)	0.79 (0.39–1.61)	0.91 (0.43–1.89)	1.07 (0.51–2.32)
10,001–15,000 (127 to 191 USD)	0.48 (0.25–0.92)*	0.47 (0.24–0.93)*	0.51 (0.25–1.03)	0.57 (0.27–1.20)
above 15,000 (above 191 USD)	0.67 (0.35–1.31)	0.81 (0.40–1.66)	0.77 (0.38–1.58)	1.05 (0.48–2.28)

^**a**^ UOR: Unadjusted odds ratio

^**b**^ AOR: Adjusted odds ratio: adjusted for age, sex, occupation, education, and monthly income

^**c**^ BDT- Bangladeshi Taka

* p<0.05

** p<0.001

Note: those with no NCD risk factors (n = 4) are excluded in the clustering analyses

## Discussion

This study provides the current evidence base in terms of NCD risk factors among the adults living in selected slum areas in Bangladesh. To the best of our knowledge, this is the first study ever being conducted to assess the NCD risk factors among this particular population living in slums and poor settlements. This study reported high prevalence of NCD risk factors among the slum adults in Bangladesh. Almost a third (30.4%) adult in slum areas had three or more NCD risk factors, which was positively associated with the younger age groups (35 to 54 years old) and no formal and below the primary level education. This proportion was slightly higher than the one reported in 2010 NCD risk factor surveys (28.3%) [22] and less than the one reported in 2014 NCD factor study (38%) [23]. The Nepal national NCD risk factor survey reported that nearly one-fifth population (18.7%) had at least three NCD risk factors [24].

The prevalence of current smoking was reported at 35.7%, which is substantially higher than the one reported in a recently conducted nationwide NCD risk factor survey in Bangladesh (17% of Bangladeshi adults as daily smokers) [23]. Our findings on current smoking is also higher than that reported in studies conducted in neighboring countries like India (14%), Nepal(19%) [24] and Sri Lanka (15%) [25]. Low level of education and lack of knowledge and awareness may be responsible for high proportion smoking. Evidence suggests that, use of tobacco in 2012 caused about six million deaths resulting from direct and second hand smoke, which is expected to increase to 7.5 million deaths per year by 2020, and will account for 10% of total deaths worldwide [26]. These findings indicate that priority should be given to exploring smoke-free options in slum areas of Dhaka, complemented by a national campaign designed to encourage smokers to quit. These interventions have been proven to be effective, with smokers who quit having their risk of myocardial infarction halved within four to five and approaching that non-smokers over a 10 to 20-year period [27, 28]. However, the benefits of smoke-free legislation go beyond reducing the risk of cardiovascular diseases to include other benefits such as improved health of general population by improving indoor air quality, reducing asthma exacerbations, and improving infant and birth outcomes [29].

The prevalence of insufficient fruits and vegetables intake among the urban slum adults was substantially high (94.9%), which is similar to the one reported in 2014 Bangladesh NCD risk factors survey (93%) [23] and this rate in Nepal was as high as99% [24]. Bangladesh in recent decades is experiencing rapid urbanization [30, 31] which also has direct impact on increased slum populations [31] and has become a major public health concern. While the country faces urbanization and related issues, easy access to low cost unhealthy food at the door steps of slum dwellers and unaffordable cost to the fruits and vegetables may be major contributors to this high proportion of people adopting unhealthy food habit thereby contributing to the increased number of people with NCD risk factors in slum areas.

Physical inactivity is also one of the major risk factors for NCDs. Worldwide, physical inactivity is responsible for at least 3.2 million deaths annually [26]. In our study, we found that 15.2% adults were physically inactive, which is corroborated to the one reported in Myanmar NCD risk factor survey (15.7%) [32], however higher than the one in Nepal (3%) [24] and much lower than the one reported by Bangladesh NCD risk factor survey (38%) [12]. This may be due to the majority of the adult slum dwellers being physically active to the manual tasks they have been engaged with. The global estimation for prevalence of physical inactivity is around 17% [33].

Obesity and overweight are modifiable risk factors to the NCDs. Our study reported the prevalence of overweight and obesity as18.7% and 3.7%, respectively. These findings are similar to the one reported in other studies including the Myanmar NCD risk factor study reported overweight of 22.3% and obesity of 5.5% [32]; Bangladesh NCD risk factor study 20.5% and 5.2%, respectively [12]; and the study in Nepal reported the combined prevalence of overweight and obesity up to 21% [24].

The self-reported diabetes prevalence was 4.9%, which is less than the one reported by the recently conducted nationwide survey in Bangladesh, that reported the age-adjusted prevalence of diabetes and pre-diabetes as 9.7% and 22.4%, respectively [34]. This may be due to self-reported from those diagnosed cases. However, there might be many slum adult dwellers who might not be aware of their diabetic conditions. A community based study conducted among adult populations in Bangladesh also reported that the prevalence of diabetes and pre-diabetes was 6.6% and 16.6%, respectively [35]. According to IDF Diabetes Atlas 5th edition, the projected prevalence of diabetes in Bangladesh will increase up to over 50% by next 15 years, placing Bangladesh as the 8^th^ranked country with the highest number of people with diabetes worldwide [36].The prevalence of hypertension as identified in this study (13.6%) was lower than the one reported by other Bangladeshi studies 26.4% [37] and 21.4% [12], and the studies conducted in neighboring countries 26.4% in Myanmar and in Nepal 26% [24].

In Bangladesh, the rapidly increasing patterns of urbanization, unplanned urban settlements and ageing population along with increased behavioral risk factors, cumulatively exacerbate the NCD situation in the country, posing further health and development consequences at individual, family and national levels [38, 39]. The increased morbidity and mortality as well as higher prevalence of NCD risk factors in Bangladesh pose significant public health concern and has greater risk of economic impact at all levels [40].

The current study has few limitations worth acknowledging. The study was conducted among the adult slum populations of selected slum areas in Dhaka, Bangladesh, which may not be representative of all the slum population across Bangladesh. Similarly, we were unable to collect biomedical data, such as blood glucose levels, and hence had to rely on self-reported diabetes, which increases the likelihood of an underestimation. Because of the cultural and religious sensitivity, we were unable to collect waist circumference measure. In this study, we found very low proportion of female currently smoking tobacco, which may be due to cultural sensitivity, such as culturally in Bangladeshi context, smoking is regarded as an unacceptable behavior and thought to be especially objectionable among females. There might be more women in slums smoking tobacco, however due to conservative societal pressures and fear of shame and dishonor may have been the reason for many women not disclosing their smoking status.

## Conclusion

This study provides the most recent and comprehensive evidence base on the status of NCD risk factors among the urban slum adult population in Dhaka Bangladesh. All NCD risk factors, in particular, smoking, physical inactivity, low consumption of vegetables and fruits, overweight and BMI, and high blood pressure are markedly high among the urban slum adults in Bangladesh. We also conclude that greater number of slum adults have at least three NCD risk factors, which was positively associated with the economically productive age groups (35 to 54 years old) and poor education levels. These findings are very timely and important to support the formulation and implementation of NCD-related polices and plan of actions that recognize urban slum populations in Bangladesh as a priority sub-population for NCDs prevention.
